# Association of Medicaid Expansion With Enrollee Employment and Student Status in Michigan

**DOI:** 10.1001/jamanetworkopen.2019.20316

**Published:** 2020-01-31

**Authors:** Renuka Tipirneni, John Z. Ayanian, Minal R. Patel, Edith C. Kieffer, Matthias A. Kirch, Corey Bryant, Jeffrey T. Kullgren, Sarah J. Clark, Sunghee Lee, Erica Solway, Tammy Chang, Adrianne N. Haggins, Jamie Luster, Erin Beathard, Susan D. Goold

**Affiliations:** 1 Institute for Healthcare Policy and Innovation, University of Michigan, Ann Arbor; 2 Department of Internal Medicine, University of Michigan, Ann Arbor; 3 Department of Health Management and Policy, School of Public Health, University of Michigan, Ann Arbor; 4 Gerald R. Ford School of Public Policy, University of Michigan, Ann Arbor; 5 Department of Health Behavior and Health Education, School of Public Health, University of Michigan, Ann Arbor; 6 School of Social Work, University of Michigan, Ann Arbor; 7 Center for Bioethics and Social Sciences in Medicine, University of Michigan, Ann Arbor; 8 Center for Clinical Management Research, Veterans Affairs Ann Arbor Healthcare System, Ann Arbor, Michigan; 9 Child Health Evaluation and Research Center, University of Michigan, Ann Arbor; 10 Institute for Social Research, University of Michigan, Ann Arbor; 11 Department of Family Medicine, University of Michigan, Ann Arbor; 12 Department of Emergency Medicine, University of Michigan, Ann Arbor

## Abstract

**Importance:**

Medicaid community engagement requirements (work, school, job searching, or community service) are being implemented by several states for the first time, but the association of Medicaid coverage with enrollees’ employment and school attendance is unclear.

**Objective:**

To assess longitudinal changes in enrollees’ employment or student status after Michigan’s Medicaid expansion.

**Design, Setting, and Participants:**

This survey study included 4090 nonelderly, adult Healthy Michigan Plan enrollees from March 1, 2017, to January 31, 2018.

**Main Outcomes and Measures:**

Self-reported employment or student status. Proportionate sampling was stratified by income and geographic region. Mixed-effects regression models with time indicators were used to assess longitudinal changes in the proportion of enrollees who were employed or students.

**Results:**

The response rate for the initial survey was 53.7% and for the follow-up survey was 83.4%. Of the 3104 respondents to the 2017 follow-up survey (mean [SD] age in 2017, 42.2 [13.0] years; 1867 [53.0%] female), 54.3% were employed or students in 2016, and this number increased to 60.0% in 2017 (percentage point change, 5.7; *P* < .001). Non-Hispanic black enrollees had significantly larger gains in employment or student status compared with non-Hispanic white enrollees (percentage point change, 10.7 vs 3.5; *P* = .02). Changes in employment or student status were not associated with improved health status.

**Conclusions and Relevance:**

Employment or student status increased from 2016 to 2017 among Michigan Medicaid expansion enrollees. These findings provide information about whether Medicaid coverage or community engagement requirements are best to promote the desired outcomes of employment and student status.

## Key Points

**Question:**

Is Medicaid expansion associated with changes in enrollees’ employment or student status?

**Findings:**

In this survey study of 4090 Michigan Medicaid expansion enrollees conducted after Michigan’s Medicaid expansion, 54.3% of respondents were employed or students in 2016 and 60.0% were employed or students in 2017.

**Meaning:**

Employment or student status increased among Michigan Medicaid expansion enrollees 2 to 3 years after the state expanded Medicaid and before implementation of community engagement requirements.

## Teaser

This survey study assesses the association of Medicaid expansion in Michigan with enrollees’ employment or student status.

## Introduction

The Centers for Medicare & Medicaid Services (CMS) has been approving community engagement requirements in state Medicaid §1115 waivers at an accelerating pace since the January 2018 announcement that supported this policy shift.^1^ With the requirement of work, school, job searching, or community service as a condition of eligibility for Medicaid, community engagement (CE) requirements have been promoted by many policy makers as a means of improving the health and financial well-being of Medicaid enrollees. As many as 11 million Medicaid enrollees who do not currently have work or other qualifying activities,^2^ ranging from approximately 2% to 10% of the nondisabled adult Medicaid population in each state,^3^ could ultimately be subject to a CE requirement. Despite ongoing legal challenges to this policy^4,5^ and concerns about its effects on enrollment of low-income individuals in Medicaid programs,^6,7,8^ CMS has approved CE requirements in 9 state waivers in the past year.^9^

Alongside this new component of Medicaid §1115 waivers, CMS has placed increasing emphasis on the importance of rigorous evaluations of these demonstration programs,^10^ including assessment of desired outcomes, such as health and employment. However, the literature to date on the association of Medicaid expansion with employment or other activities that could qualify for CE has been limited. Several national studies^11,12,13,14^ have observed no overall change in employment or working hours after Medicaid expansion, whereas 1 study^15^ found increased rates of volunteer activities in Medicaid expansion states. However, none of these studies examined actual Medicaid enrollees.

Studies^16,17^ of specific populations or states have suggested that Medicaid expansion might be associated with increased employment. For example, among individuals with disabilities, Medicaid expansion was associated with increased employment rates. Cross-sectional studies of Medicaid expansion enrollees’ perspectives in Michigan^18^ and Ohio^19^ have recently demonstrated positive associations with health and ability to work or seek employment. However, no prior studies, to our knowledge, have assessed longitudinal changes in employment or other CE activities among Medicaid enrollees.

As more states implement CE requirements in 2020 and beyond, it is important to understand the patterns of employment and other qualifying activities, such as school enrollment, among Medicaid enrollees in the absence of such requirements. The present study evaluated Michigan’s Medicaid expansion, known as the Healthy Michigan Plan (HMP), which has expanded coverage to approximately 680 000 low-income individuals and has been approved by CMS to implement a CE requirement in 2020. The study objective was to assess longitudinal changes in enrollees’ employment or student status after HMP enrollment.

## Methods

### Study Design

For this study, we conducted a structured telephone survey in English, Arabic, and Spanish of 4090 HMP enrollees from January 1 to October 31, 2016, and a follow-up survey of 3104 of these respondents from March 1, 2017, to January 31, 2018, as part of an evaluation of HMP under contract with the Michigan Department of Health and Human Services. Institutional review boards from the University of Michigan and Michigan Department of Health and Human Services deemed this evaluation of a public program to be exempt from review; therefore, no informed consent was required. Data were not deidentified. This study followed the American Association for Public Opinion Research (AAPOR) reporting guideline.

We used the AAPOR response rate formula 3.^20^ Methods of the 2016 survey have been described previously.^18,21^ For the 2017 follow-up survey, 2 instruments were developed: 1 for those who remained enrolled in HMP and 1 for those who were no longer enrolled at the time of the survey.

### Survey Sampling and Administration

Sampling was stratified by income and geographic region. Eligible enrollees for the 2016 survey were aged 19 to 64 years with 12 months or more of HMP coverage and 9 months or more in a Medicaid health plan. Those who agreed to be contacted for follow-up (3957 [96.4%] of 2016 respondents) composed the 2017 sample.

Introductory packets that described the 2017 follow-up survey were mailed to 2016 survey respondents approximately 13 to 14 months after completion of the 2016 survey; additional reminders were sent by email or text message to those who provided contact information. Survey interviewers then called individuals and recorded responses using computer-assisted telephone interviewing software (CATI software; Voxco). Respondents were mailed a $25 gift card after completion of the survey.

### Measures

In 2016, a single item asked, “What is your current job status? Are you currently…?” with respondents asked to select 1 of the following response options: employed or self-employed, out of work (≥1 year), out of work (<1 year), homemaker, student, retired, or unable to work. In 2017, items were asked separately: (1) “Are you currently in school?” (yes or no) and (2) “Are you currently employed or self-employed?” (yes or no). For those who responded that they were not employed, a follow-up question asked, “Are you out of work, unable to work, retired, or not looking for work at this time?”

Health status was assessed in both 2016 and 2017 by the question, “In general, would you say your health is…?” with the following response options: excellent, very good, good, fair, or poor. Improved health status was defined as reporting fair or poor health in 2016 and then reporting excellent, very good, or good health in 2017.

Most demographic characteristics, including age, sex, income, and geographic region, were obtained from Medicaid program files in 2016. Self-reported race/ethnicity was obtained from the 2016 survey, and educational attainment was obtained from the 2017 survey. Surveys included standard measures of health characteristics from established national surveys.^22,23,24,25,26^

### Subgroup Identification

We also examined trends in outcomes among vulnerable subgroups. Enrollees with 1 or more chronic physical health conditions were identified using Medicaid claims in the 24-month period before survey sampling and the 12-month period after survey sampling and/or by self-report in the 2016 or 2017 survey. Enrollees with 1 or more mental health or substance use disorders were identified using Medicaid claims in the 24-month period before survey sampling and the 12-month period after survey sampling.

### Benchmarking of HMP Survey Findings Against Statewide Trends

Comparison measures of statewide changes in employment or student status were obtained from the US Census Bureau Current Population Survey (CPS) Annual Social and Economic Supplement, 2016 and 2017. Respondents were considered to be employed if they were civilians who at the time of survey reported doing any work as paid employees, were self-employed in their own business or farm, worked at least 15 hours as an unpaid worker at a business or on a farm owned by a family member, or had a job but were not currently working because of illness, weather, vacation, time off for personal reasons, or labor-management disputes. Respondents were considered to be students if they were enrolled in school full-time or part-time and had attended within the past week. Employed and student categories were combined, and the method of Lee et al^27^ for examining trends in serial cross-sectional survey data was used to compare 2016 and 2017 data. We evaluated trends among all nonelderly adults and among adults with incomes similar to those eligible for Medicaid (income ≤138% of the federal poverty level [FPL]) using a low-income threshold of 125% FPL available in the CPS. All analyses incorporated CPS survey weights.

### Statistical Analysis

Survey weights were calculated and applied to all analyses to adjust for sample design, nonresponse bias, and poststratification adjustments. Descriptive statistics are presented as weighted percentages. We used mixed models with survey year as a fixed effect and individual respondent as a random effect to assess longitudinal changes in the proportion of enrollees who were employed or students.^28^ Mixed-effects logistic regression models were used to identify variables associated with being employed or a student, adjusting for age, sex, race/ethnicity, income, educational level, survey year, and health status. We also assessed interactions between demographic factors (age, sex, and race/ethnicity) and period to determine whether changes in employment or student status varied significantly by these groups. All analyses were conducted with Stata software, version 15 (StataCorp), with 2-sided *P* < .05 considered to be statistically significant.

### Sensitivity Analyses

To assess whether employment or student status would have differed between 2017 survey respondents and nonrespondents, we examined 2016 employment or student status. Using 2016 data, we also conducted multiple imputation analysis estimating 2017 employment or student status that was missing for 2017 survey nonrespondents.

## Results

### Survey Respondents’ Demographic Characteristics

The response rate for the initial survey was 53.7% and for the follow-up survey was 83.4%. We observed little difference between those who consented to be recontacted and those who did not (eTable 1 in the Supplement). Among those who consented to be recontacted, analysis of respondents vs nonrespondents showed slight differences. Respondents to the 2017 follow-up survey were more likely to be older, have lower income, and be English speaking (eTable 2 and eTable 3 in the Supplement).

A total of 3104 enrollees responded to the 2017 follow-up survey (mean [SD] age in 2017, 42.2 [13.0] years; 1867 [53.0%] female). At the time of the 2017 survey, 76.8% (95% CI, 74.9%-78.6%) were still enrolled in HMP, and 23.2% (95% CI, 21.4%-25.1%) were no longer enrolled (Table 1). A total of 52.3% (95% CI, 51.2%-53.5%) had incomes of 0% to 35% of the FPL, and 80.9% (95% CI, 79.7%-82.0%) lived in urban regions.

**Table 1.** Demographic Characteristics of 2017 Follow-up Survey Respondents

Characteristic	Respondents, % (95% CI) (N = 3104)^a^
Enrollment status at time of follow-up survey	
Still enrolled	76.8 (74.9-78.6)
No longer enrolled	23.2 (21.4-25.1)
Age group, y^b^	
19-34	37.5 (35.3-39.9)
35-50	34.0 (31.8-36.2)
51-64	28.5 (26.7-30.3)
Sex^b^	
Male	47.0 (44.7-49.2)
Female	53.0 (50.8-55.3)
Race/ethnicity^c^	
White, non-Hispanic	59.6 (57.4-61.7)
Black, non-Hispanic	26.8 (24.8-28.9)
Hispanic	5.0 (4.1-6.0)
Other, non-Hispanic	8.7 (7.4-10.1)
FPL category, %^b^	
0-35	52.3 (51.2-53.5)
36-99	27.7 (26.8-28.7)
100-133	19.9 (19.2-20.7)
Highest level of education^d^	
Less than high school	11.2 (9.9-12.6)
High school graduate	40.5 (38.3-42.8)
Some college	22.8 (20.9-24.9)
Associate’s degree	12.9 (11.5-14.5)
Bachelor’s degree	9.9 (8.6-11.3)
Postgraduate degree	2.7 (2.0-3.5)
Urbanicity^b^	
Urban	80.9 (79.7-82.0)
Suburban	8.9 (7.9-10.0)
Rural	10.2 (9.6-10.8)
Geographic region^b^	
Northern Michigan	9.1 (8.7-9.5)
Central Michigan	29.6 (28.6-30.6)
Southern Michigan	18.4 (17.6-19.3)
Detroit metropolitan area	42.9 (41.7-44.1)

Abbreviation: FPL, federal poverty level.

^a^ Percentages are weighted.

^b^ Variable from 2016 Medicaid Claims Data Warehouse.

^c^ Variable from 2016 survey.

^d^ Variable from 2017 survey.

### Changes in Enrollee Employment or Student Status

Among all respondents, 54.3% were employed or students in 2016, and this percentage increased to 60.0% in 2017 (percentage point change, 5.7; *P* < .001) (Figure, A). Among those still enrolled in HMP in 2017, the number who were employed or students increased from 53.1% to 58.7% (percentage point change, 5.6; *P* < .001); among those no longer enrolled in 2017, the proportion who were employed or students increased from 58.2% to 64.1% (percentage point change, 5.9; *P* = .002). Increases in employment or student status from 2016 to 2017 were also noted among subgroups of enrollees with chronic health conditions (percentage point change, 6.5; *P* < .001) and enrollees with mental health or substance use disorders (percentage point change, 6.1; *P* < .001).

**Figure.**
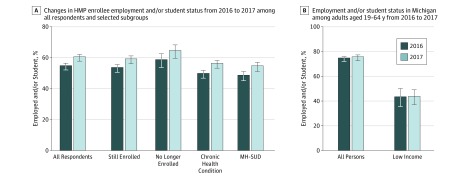
Changes in Enrollee Employment or Student Status From 2016 to 2017 Among All Respondents and Selected Subgroups A, Persons with chronic health conditions included survey respondents with 1 claims-based diagnosis of a chronic physical health condition. Mental health or substance use disorder (MH-SUD) included survey respondents with 1 claims-based diagnosis of a chronic behavioral health condition, including mental health disorders and/or substance use disorders. B, Low income is defined as persons whose household income is at or below 125% of the federal poverty level.This census category approximates Healthy Michigan Plan (HMP) income eligibility, which is 138% of the federal poverty level. Employed and/or student includes those who are employed or are full-time or part-time students. Weighted percentages are presented. Error bars indicate 95% CIs. *P* < .05 for employment or student status change among HMP enrollees from 2016 to 2017 for all comparisons, estimated from mixed-effects logistic regression model with time indicators but no covariates. *P* > .05 for comparisons among adults aged 19 to 64 years in Michigan.

Enrollees who were 35 to 50 years old (percentage point change, 8.0) and 51 to 64 years old (percentage point change, 5.7), male (percentage point change, 6.7), non-Hispanic black (percentage point change, 10.7), and in the lowest income category (percentage point change, 9.2) had larger increases in employment or student status (Table 2). Enrollees in Northern Michigan, the most rural region of the state, had smaller increases in employment or student status compared with other regions (percentage point change, 2.2; compared with up to 6.2 in other regions).

**Table 2.** Employment or Student Status Change From 2016 to 2017 by Selected Demographic Characteristics

Characteristic	Employed or Student, % (95% CI)^a^		Difference in Percentage Points
	2016	2017	
Age group, y^b^			
19-34	70.0 (66.2-73.7)	73.5 (69.9-77.1)	3.5
35-50	49.8 (45.8-53.7)	57.8 (53.9-61.7)	8.0
51-64	39.0 (35.8-42.3)	44.7 (41.3-48.1)	5.7
Sex^b^			
Male	52.2 (48.7-55.7)	58.9 (55.5-62.4)	6.7
Female	56.1 (53.3-59.0)	60.9 (58.1-63.6)	4.8
Race/ethnicity^c^			
White, non-Hispanic	54.0 (51.3-56.7)	57.5 (54.8-60.2)	3.5
Black, non-Hispanic	51.0 (46.2-55.9)	61.7 (57.1-66.3)	10.7
Hispanic	68.7 (60.0-77.4)	71.5 (63.2-79.8)	2.8
Other, non-Hispanic	60.2 (52.6-67.9)	65.8 (58.5-73.2)	5.6
FPL category, %^b^			
0-35	38.2 (34.6-41.8)	47.4 (43.8-51.0)	9.2
36-99	69.2 (66.0-72.4)	72.8 (69.8-75.7)	3.6
100-133	75.6 (72.4-78.9)	75.0 (71.7-78.3)	−0.6
Geographic region^b^			
Northern Michigan	55.5 (51.1-59.9)	57.7 (53.3-62.1)	2.2
Central Michigan	51.1 (47.7-54.6)	57.3 (53.9-60.8)	6.2
Southern Michigan	56.9 (52.5-61.2)	62.7 (58.4-67.0)	5.8
Detroit metropolitan area	55.1 (51.1-59.1)	61.1 (57.2-65.0)	6.0

Abbreviation: FPL, federal poverty level.

^a^ Percentages are weighted.

^b^ Variable from 2016 Medicaid Claims Data Warehouse.

^c^ Variable from 2016 survey. In interaction analyses, there was no statistically significant difference in observed changes across age or sex groups. Non-Hispanic black enrollees had significantly larger gains in employment or student status compared with the non-Hispanic white reference group (percentage point change, 10.7 vs 3.5; *P* = .02); changes for Hispanic ethnicity (percentage point change, 2.8; *P* = .94) and other race/ethnicity (percentage point change, 5.6; *P* = .54) groups were not significantly different compared with the reference group.

In interaction analyses between demographic factors and analysis period, no statistically significant differences across age or sex groups were found. However, non-Hispanic black individuals had significantly larger gains in employment or student status compared with a non-Hispanic white reference group (percentage point change of 10.7 among non-Hispanic black individuals vs. 3.5 in the reference group, *P* = .02) (Table 2). Changes for Hispanic ethnicity (percentage point change, 2.8; *P* = .94) and other race/ethnicity groups (percentage point change, 5.6; *P* = .54) were not significantly different compared with the reference group (percentage point change, 3.5).

### Variables Associated With Employment or Student Status

In mixed-effects regression models that assessed variables associated with employment or student status, respondents were more likely to be employed or students if they were non-Hispanic black or Hispanic compared with non-Hispanic white or had higher educational attainment compared with high school or less (eTable 4 in the Supplement). Variables associated with employment or student status were largely similar across prespecified subgroups of enrollees with chronic health conditions and enrollees with mental health or substance use disorders (eTable 5 in the Supplement).

### Association Between Employment or Student Status and Changes in Health

Employment or student status in 2017 was not significantly associated with improved health status (adjusted odds ratio, 1.16; 95% CI, 0.65-2.08; *P* = .61), after adjusting for age, sex, race/ethnicity, FPL, and educational level.

### Benchmarking Against Statewide Trends in Employment or Student Status

Among all individuals aged 19 to 64 years in Michigan, 74.0% were employed or students in 2016 and 75.0% were employed or students in 2017 (percentage point change, 1.0; *P* = .64) (Figure, B). Among low-income residents aged 19 to 64 years, 42.7% were employed or students in 2016 and 43.0% were employed or students in 2017 (percentage point change, 0.3; *P* = .95). Although HMP enrollees experienced significant gains in employment or student status between 2016 and 2017, employment or student status trends in the state remained unchanged (Figure).

### Sensitivity Analysis

 When we assessed for differences between 2017 respondents and nonrespondents, we found no statistically significant differences in 2016 employment or student status between the 2 groups. With imputed data estimating employment or student status that was missing for 2017 nonrespondents, there were still no statistically significant differences in employment or student status between 2017 respondents and nonrespondents.

## Discussion

In this longitudinal survey study of Medicaid expansion enrollees in Michigan, we found that employment or student status increased from 2016 to 2017, approximately 2 to 3 years after implementation of the program. This increase occurred among those who were still enrolled and those no longer enrolled in HMP in 2017. Non-Hispanic black enrollees exhibited the greatest gains in employment or student status during this period. These increases in employment or student status among HMP enrollees occurred while statewide levels remained unchanged. To our knowledge, this is the first study to assess longitudinal changes in elements of community engagement in Medicaid expansion enrollees.

Few other studies have examined the association of Medicaid expansion with community engagement, and most of these have focused on employment. Although the Medicaid expansion program in Ohio was associated with improvements in ease of working or looking for work^19^ and another study^16,17^ found that people with disabilities were more likely to be employed after Medicaid expansion, most studies^11,12,13,14,29^ have not observed changes in employment associated with Medicaid expansion. In one of the only experimental studies of Medicaid (the Oregon Health Insurance Experiment),^30^ there was no significant effect of being randomized to Medicaid coverage on employment or earnings. On the one hand, it is possible that these differences in the literature relate to differences in study design. Our longitudinal assessment of Medicaid expansion enrollees may be more likely to detect changes in employment or student status than studies of individuals who are estimated to be Medicaid eligible but may not actually be enrolled (eg, secondary analyses of federal surveys). On the other hand, this finding may reflect new observations of employment changes attributable to longer-term effects of Medicaid expansion that were not evident in earlier periods. Because much of the study population had been enrolled for more than 2 years, it is possible that gains in employment or student status associated with Medicaid expansion take time to manifest and may continue to increase in the future.

Positive gains in employment or student status were most prominent among racial/ethnic minority groups, who have historically faced discrimination in the labor market.^31,32^ Similar socioeconomic consequences for persons of racial/ethnic minority groups have been associated with other social safety-net programs, such as the Temporary Assistance for Needy Families program. Following implementation of Temporary Assistance for Needy Families, black and Hispanic families had large gains in household income,^33^ including after exit from the program.^34^

Although a previous cross-sectional study^18^ suggested a positive association between enrollee reports of health improvement and job-related outcomes, the present longitudinal study found no significant association of changes in employment or student status with changes in health status. Nonetheless, we still found evidence of substantial gains in employment or student status among subgroups at risk of poor health, including those with chronic conditions or behavioral health disorders. Although improvements in ability to work and job seeking associated with improved health were previously reported,^18^ it appears that changes in overall employment or student status may have occurred through a different mechanism. It could be that after obtaining health insurance, which protects against financial losses, enrollees invested greater resources in other areas to improve their success in gaining employment or attending school.

Regardless of the mediators of the observed gains in employment or student status among Medicaid expansion enrollees, the study findings are salient for the current policy discussion about CE requirements in states across the country. The federal government is currently encouraging states to experiment with these requirements to promote employment and health among Medicaid enrollees. Our findings raise the question of whether Medicaid coverage or CE requirements are the best path to promoting the desired outcomes of employment and student status. We believe that states should consider these implications as they decide whether or how to implement CE requirements in their Medicaid programs.

### Limitations

Our study has limitations. First, responses may reflect social desirability or recall bias potentially present in all surveys. However, measures were drawn from large national surveys and thus are consistent with established methods. Second, there is the potential for bias because only those who consented to follow-up were sampled. However, our analysis indicated little difference between those who did and did not consent to be recontacted. Nonresponse bias could also influence the results, although we did not find many differences between respondents and nonrespondents, and we applied weights to adjust for such differences. Third, employment and student status were assessed in a single item in 2016 and in 2 separate items in 2017. Because our focus was on CE-qualifying activities broadly, our primary outcome was defined as combined employment and student statuses. Fourth, because we did not have a baseline assessment before expansion in 2014, it is possible that the present study did not account for earlier changes and may have missed unstable employment or student status history before 2016 or underestimated the association of Medicaid coverage with employment or student status if earlier positive gains in these outcomes were present. Fifth, we did not assess other activities that would qualify as community engagement, such as job searching, job training, or volunteering. Sixth, causal inference could not be determined for this observational study. We assessed statewide temporal trends to provide a benchmark for observed changes in outcomes.

## Conclusions

This study found that employment or student status increased from 2016 to 2017 among Michigan Medicaid expansion enrollees. These findings provide information about whether Medicaid coverage or CE requirements are best to promote the desired outcomes of employment and student status.

## Notes

Supplement. **eTable 1.** Comparison of 2016 Enrollee Survey Respondents Who Consented to Be Recontacted and Those Who Did Not **eTable 2.** Comparison of Characteristics of 2017 Enrollee Follow-up Survey Respondents and Nonrespondents **eTable 3.** 2017 Enrollee Survey Responses Among 2016 Respondents Who Consented to Be Recontacted **eTable 4.** Predictors of Employment of Student Status Among All Respondents, Multivariable Mixed Effects Regression **eTable 5.** Change in Employment or Student Status Between 2016 And 2017 Among Subgroups, Multivariable Mixed Effects Regression Adjusted for Demographics and Health Status
