# Supplementary material for: Association of Medicaid Expansion With Enrollee Employment and Student Status in Michigan
Source: JAMA Netw Open. 2020 Jan 31;3(1):e1920316. doi: 10.1001/jamanetworkopen.2019.20316 (PMC7042869; doi:10.1001/jamanetworkopen.2019.20316)
Supplement: Supplement. — eTable 1. Comparison of 2016 Enrollee Survey Respondents Who Consented to Be Recontacted and Those Who Did Not eTable 2. Comparison of Characteristics of 2017 Enrollee Follow-up Survey Respondents and Nonrespondents eTable 3. 2017 Enrollee Survey Responses Among 2016 Respondents Who Consented to Be Recontacted eTable 4. Predictors of Employment of Student Status Among All Respondents, Multivariable Mixed Effects Regression eTable 5. Change in Employment or Student Status Between 2016 And 2017 Among Subgroups, Multivariable Mixed Effects Regression Adjusted for Demographics and Health Status [file jamanetwopen-3-e1920316-s001.pdf]

Supplementary Online Content

Tipirneni R, Ayanian JZ, Patel MR, et al. Association of Medicaid expansion with enrollee employment and student status in Michigan. *JAMA Netw Open*. 2020;3(1):e1920316. doi:10.1001/jamanetworkopen.2019.20316

eTable 1. Comparison of 2016 Enrollee Survey Respondents Who Consented to Be Recontacted and Those Who Did Not

eTable 2. Comparison of Characteristics of 2017 Enrollee Follow-up Survey Respondents and Nonrespondents

eTable 3. 2017 Enrollee Survey Responses Among 2016 Respondents Who Consented to Be Recontacted

eTable 4. Predictors of Employment of Student Status Among All Respondents, Multivariable Mixed Effects Regression

eTable 5. Change in Employment or Student Status Between 2016 And 2017 Among Subgroups, Multivariable Mixed Effects Regression Adjusted for Demographics and Health Status

This supplementary material has been provided by the authors to give readers additional information about their work.

© 2020 Tipirneni R et al. *JAMA Network Open.*

eTable 1. Comparison of 2016 enrollee survey respondents who consented to be recontacted and those who did not

Characteristics	Consented to be recontacted N=3,957 %	Did not consent to be recontacted N=149 %	*p* value
Age			
19-34	31.7%	36.2%	0.16
35-50	31.7%	34.9%	
51-64	36.6%	28.9%	
Gender			
Male	40.7%	53.3%	0.002
Female	59.3%	46.7%	
Race/ethnicity			
Non-Hispanic White	33.8%	37.3%	0.37
Other	66.2%	62.7%	
Income (FPL category)			
0-35% FPL	39.2%	38.3%	0.62
36-99% FPL	35.3%	38.9%	
100-133% FPL	25.5%	22.8%	
Geographic region			
Northern Michigan	18.3%	14.8%	0.34
Central Michigan	31.0%	27.5%	
Southern Michigan	20.4%	21.5%	
Detroit Metro	30.2%	36.2%	
Interview language			
Arabic	1.5%	0.7%	0.47
English	97.9%	99.3%	
Spanish	0.5%	0%	

© 2020 Tipirneni R et al. *JAMA Network Open.*

eTable 2. Comparison of characteristics of 2017 enrollee follow-up survey respondents and nonrespondents

Characteristics	Respondents N=3,104 %	Nonrespondents N=608 %	*p* value
Age[a]			
19-34	40.1%	49.7%	<0.001
35-50	29.5%	30.0%	
51-64	30.5%	20.3%	
Gender[a]			
Male	47.2%	48.7%	0.61
Female	52.8%	51.3%	
Race/ethnicity[b]			
Non-Hispanic White	60.3%	60.8%	0.88
Other	39.7%	39.2%	
FPL category[a]			
0-35% FPL	53.1%	45.6%	0.02
36-99% FPL	27.7%	32.1%	
100-133% FPL	19.1%	22.3%	
Interview language[b]			
Arabic	0.8%	5.3%	<0.001
English	98.7%	93.6%	
Spanish	0.5%	1.2%	
Geographic region[a]			
Northern Michigan	8.9%	9.4%	0.25
Central Michigan	29.3%	24.8%	
Southern Michigan	18.1%	20.5%	
Detroit Metro	43.7%	45.3%	

Authors' analysis of data from the 2016 Healthy Michigan Voices Survey and the 2017 Healthy Michigan Voices Follow-Up Survey. Chi-square test of independence. [a]Variable from 2016 Medicaid Claims Data Warehouse, [b]Variable from 2016 survey.

© 2020 Tipirneni R et al. *JAMA Network Open.*

eTable 3. 2017 enrollee survey responses among 2016 respondents who consented to be recontacted

Call Results	N	%
Response	3104	75.6
Nonresponse	608	14.8
Partial complete	6	0.1
Refusal	166	4.0
Noncontact/Other nonresponse	436	10.6
Ineligible	394	9.6
Not the correct number	105	2.6
Deceased	50	1.2
Unable to complete in English, Spanish or Arabic	2	0.0
Non-working phone number	88	2.1
Did not consent in 2016 to follow-up contact	149	3.6
Total	4,106	100.0

© 2020 Tipirneni R et al. *JAMA Network Open.*

eTable 4. Predictors of employment or student status among all respondents, multivariable mixed effects regression

	aOR	95% CI
Survey year		
2016	Ref	
2017	1.7	(1.4, 2.2)
Age[a]		
19-34	Ref	
35-50	0.3	(0.2, 0.4)
51-64	0.1	(0.1, 0.1)
Gender[a]		
Male	Ref	
Female	0.8	(0.6, 1.2)
Race/ethnicity[b]		
White, non-Hispanic	Ref	
Black, non-Hispanic	1.7	(1.2, 2.6)
Hispanic	2.9	(1.3, 6.5)
Other, non-Hispanic	1.9	(1.0, 3.7)
FPL category[a]		
0-35%	Ref	
36-99%	15.1	(9.3, 24.6)
100-133%	23.1	(13.4, 39.8)
Highest level of education[c]		
High school or less	Ref	
Associate's degree/some college	3.2	(2.1, 4.7)
Bachelor's/post-graduate degree	4.8	(2.7, 8.6)
Fair/poor health status[d]		
No	Ref	
Yes	0.2	(0.2, 0.3)

Authors' analysis of data from the 2016 Healthy Michigan Voices Survey and the 2017 Healthy Michigan Voices Follow-Up Survey. Mixed effects logistic regression. [a]Variable from 2016 Medicaid Claims Data Warehouse, [b]Variable from 2016 survey, [c]Variable from 2017 survey, [d]Variable from both 2016 and 2017 surveys. In analyses including an interaction between race/ethnicity and time period, non-Hispanic Black respondents had greater odds of employment or student status compared with non-Hispanic Whites (aOR 2.1, *p*=0.013).

© 2020 Tipirneni R et al. *JAMA Network Open.*

eTable 5. Change in employment or student status between 2016 and 2017 among subgroups, multivariable mixed effects regression adjusted for demographics and health status

	Chronic disease[a]	MH-SUD[b]
	aOR (95% CI)	aOR (95% CI)
Survey year		
2016	Ref	Ref
2017	1.9 (1.5, 2.4)	1.8 (1.4, 2.5)
Age[c]		
19-34	Ref	Ref
35-50	0.3 (0.2, 0.5)	0.2 (0.1, 0.4)
51-64	0.1 (0.1, 0.2)	0.1 (0.0, 0.2)
Gender[c]		
Male	Ref	Ref
Female	0.9 (0.6, 1.3)	1.4 (0.9, 2.2)
Race/ethnicity[d]		
White, non-Hispanic	Ref	Ref
Black, non-Hispanic	2.0 (1.3, 3.2)	1.6 (0.9, 2.9)
Hispanic	2.8 (1.0, 7.4)	3.9 (1.3, 11.6)
Other, non-Hispanic	1.8 (0.8, 4.1)	1.5 (0.6, 3.6)
FPL category[c]		
0-35%	Ref	Ref
36-99%	16.3 (9.2, 28.9)	14.0 (7.2, 27.3)
100-133%	28.9 (15.1, 55.3)	19.3 (9.2, 40.4)
Highest level of education[e]		
High school or less	Ref	Ref
Associate's degree/some college	3.1 (2.0, 4.9)	3.0 (1.8, 5.1)
Bachelor's/post-graduate degree	4.3 (2.2, 8.4)	4.0 (1.8, 8.8)
Fair/poor health status[f]		
No	Ref	Ref
Yes	0.3 (0.2, 0.4)	0.2 (0.1, 0.3)

Authors' analysis of data from the 2016 Healthy Michigan Voices Survey and the 2017 Healthy Michigan Voices Follow-Up Survey. Mixed effects logistic regression. [a]Respondents with ≥1 claims-based diagnosis of a chronic physical health condition, [b]MH-SUD= Respondents with ≥1 claims-based diagnosis of a mental health or substance use disorder, [c]Variable from the 2016 Medicaid Claims Data Warehouse, [d]Variable from 2016 survey, [e]Variable from 2017 survey, [f]Variable from both 2016 and 2017 surveys.

© 2020 Tipirneni R et al. *JAMA Network Open.*
